# Circ-CDK8 regulates SLC7A11-mediated ferroptosis by inhibiting miR-615-5p to promote progression in oral squamous cell carcinomas

**DOI:** 10.3389/fphar.2024.1432520

**Published:** 2024-08-07

**Authors:** Kai Sun, Ling Gao, Shaoming Li, Jingjing Zheng, Zhuang Zhu, Keqian Zhi, Wenhao Ren

**Affiliations:** ^1^ Department of Oral and Maxillofacial Reconstruction, The Affiliated Hospital of Qingdao University, Qingdao, China; ^2^ School of Stomatology, Qingdao University, Qingdao, China; ^3^ Department of Oral and Maxillofacial Surgery, The Affiliated Hospital of Qingdao University, Qingdao, China; ^4^ Key Lab of Oral Clinical Medicine, The Affiliated Hospital of Qingdao University, Qingdao, China; ^5^ Department of Endodontics, The Affiliated Hospital of Qingdao University, Qingdao, China

**Keywords:** oral squamous cell carcinoma, circ-CDK8, SLC7A11, ferroptosis, migration, invasion

## Abstract

**Introduction:** Ferroptosis is a new mode of programmed cell death distinct from necrosis, apoptosis, and autophagy, induced by iron-ion-dependent lipid peroxide accumulation. Circular RNAs are a class of endogenous non-coding RNAs that regulate the biological behavior of tumors. However, the role of circ-CDK8 in regulating ferroptosis, migration, and invasion of oral squamous cell carcinoma (OSCC) remains unknown.

**Methods:** The effect of circ-CDK8 on OSCC cell ferroptosis, migration, and invasion was evaluated using CCK-8, wound healing, transwell, reactive oxygen species (ROS), malondialdehyde (MDA), and GSH assays and Western blotting. Bioinformatics analyses and luciferase reporter assays were performed and revealed targeted relationships between circ-CDK8 and miR-615-5p, miR-615-5p and SLC7A11. Interference with circ-CDK8 expression reduced SLC7A11 expression by sponging miR-615-5p, suppressed OSCC cell migration and invasion, and promoted ferroptosis by increasing ROS, MDA, and iron levels and decreasing GSH and GPX4 levels in OSCC cells. Furthermore, *in vivo,* animal experiments confirmed that circ-CDK8 interference inhibited OSCC cell proliferation and SLC7A11 expression.

**Results:** Collectively, this study revealed a novel strategy to upregulate erastin-induced ferroptosis in OSCC cells via the circ-CDK8/miR-615-5p/SLC7A11 axis, providing new insights into OSCC and a potential therapeutic strategy for OSCC.

## Introduction

Oral squamous cell carcinoma (OSCC) is the most common head and neck malignant tumor (Duprez et al., 2017). It is characterized by high morbidity and mortality, with 1,958,310 new cases and 609,820 deaths in the United States (Siegel et al., 2023). Despite recent advances, the treatment of OSCC is still based on a combination of surgery, followed by radiotherapy, chemotherapy, and immunotherapy, with a 5-year survival rate of approximately 40%–60% (Mody et al., 2021). However, patients who cannot tolerate surgery and have high facial appearance requirements cannot benefit from this treatment (Ringash, 2015), and the side effects, poor clinical response, and other problems related to radiotherapy and chemotherapy affect their clinical application (Meric-Bernstam et al., 2021). Therefore, identifying the potential carcinogenic mechanism of OSCC and specific therapeutic targets for OSCC is warranted.

Circular RNAs (circRNAs) are a class of endogenous non-coding RNAs that produce covalent closed-loop structures by back splicing. They have been shown to regulate the biological behavior of tumors (Li et al., 2020b; Chen, 2020; Zhao et al., 2020; Zheng et al., 2022; Cui et al., 2023). Thus, in our previous study, we used circRNA microarrays to screen for differential circRNA expression profiles in paired OSCC and normal tissues and found a clinically significant downregulation of the circRNA circ-PKD2 and an upregulation of the circRNA circ-CDK8 (Gao et al., 2019). The study showed that circ-PKD2 can promote autophagy by targeting miR-646, which promotes the sensitivity of OSCC cells to cisplatin chemotherapy (Gao et al., 2019; Gao et al., 2022). Therefore, we intend to explore the regulatory mechanism of circ-CDK8 in OSCC.

In 2012, ferroptosis was reported as a novel form of iron-dependent cell death (Dixon et al., 2012). It was primarily categorized into two pathways: endogenous and exogenous (Li et al., 2020a). The exogenous route is mainly concerned with the inhibition of the glutamate/cystine antiporter system (system Xc^−^), as well as the activation of iron transporters such as serotransferrin and lactotransferrin (Chen et al., 2021). The endogenous pathway involves the suppression of the biosynthesis of the intracellular antioxidant enzyme, glutathione peroxidase 4 (GPX4). Drugs like Erastin and RSL3 induce ferroptosis in RAS-deficient cells via these endogenous and exogenous pathways, respectively (Yang and Stockwell, 2008). The main characteristics of ferroptosis include the accumulation of lipid peroxides, muscle building of reactive oxygen species (ROS), and the accumulation of iron ions (Liang et al., 2019; Chen et al., 2021). Cell ultrastructure showed that ferroptosis with mitochondrial atrophy, shrinkage or disappearance of mitochondrial ridges, increased membrane density, and normal nuclear morphology but lack of chromatin agglutination (Gan, 2021; Tan et al., 2022).

Increased intracellular oxygen elevates the levels of harmful products, particularly reactive oxygen species (ROS), triggering cell death (Conrad and Pratt, 2019). Research has indicated that GPX4 and ferroptosis suppressor protein 1 (FSP1) inhibited the accumulation of ROS and the progression of ferroptosis. Consequently, the occurrence of ferroptosis is influenced by three key pathways: (1) the system Xc^−^/GPX4-related pathway, (2) the FSP1-related pathway, and (3) the iron metabolism pathway. Targeting ferroptosis associated pathways in cancer cells can be an effective strategy for cancer therapy. For example, *Shen* et al. found that Poly (rC)-binding Protein 2 (PCBP2), an RNA-binding protein, could bind and stabilize the expression of SLC7A11 mRNA, which inhibiting tumor ferroptosis and promoting tumor progression in bladder cancer (Shen et al., 2022). Sun et al. demonstrated that NF-kB activating protein (NKAP) protected glioblastoma cells from ferroptosis by promoting SLC7A11 mRNA splicing in an m^6^A-dependent manner (Sun K. et al., 2022). We previously demonstrated that SLC7A11 is a key protein that regulates ferroptosis (Sun S. et al., 2022). Besides, as an inducer of iron death sorafenib and sulfasalazine have been widely used in the targeted therapy of clinical cancer (Chen et al., 2021). So ferroptosis will be an interesting option for studies. Additionally, we found that circ-CDK8 was upregulated in OSCC cells and tissues. To further assess the interaction between circ-CDK8 and SLC7A11, we analyzed the starBase v2.0 and TargetScan databases and found that miR-615-5p served as a bridge. Therefore, this study aims to confirm that circ-CDK8 regulates the biological behavior of OSCC via ferroptosis.

In this study, the expression of circ-CDK8 in 43 pairs of OSCC tissues and paired normal tissues was analyzed. We found that circ-CDK8 was highly expressed in OSCC tissues. We report for the first time that circ-CDK8 regulates ferroptosis through the miR-615-5p/SLC7A11 axis. Further functional experimental further demonstrated that downregulation of circ-CDK8 transcription inhibited the proliferation, migration and invasion of OSCC cells. Therefore, this study provides a new potential therapeutic target for OSCC treatment.

## Materials and methods

### Cell culture and transfection

Cell lines SCC-25, CAL-27, and HOK were purchased from the Cell Bank of Type Culture Collection of the Chinese Academy of Science (Shanghai, China). The cells were cultured in Dulbecco’s modified Eagle’s medium (DMEM, Pricella, China) supplanted with 10% fetal bovine serum (FBS, HyClone, United States) and a mixture of 1% penicillin-streptomycin (Pricella, China) at 37°C under 5% CO_2_ atmosphere.

Si-circ-CDK8, miR-615-5p inhibitor, si-SLC7A11, and negative control (NC) plasmid were purchased from GenePharma (Shanghai, China). The cells were inoculated in a six-well plate and transfected with Lipofectamine 3,000 (Thermo Fisher Scienticic) when the cell density reached 60%.

Ferrostain-1 (Selleck, S7243), a potent and selective ferroptosis inhibitor, Z-VAD-FMK (Selleck, S7023), a cell permeable, irreversible caspase inhibitor, and Necrosulfonamide, a specific and effective inhibitor of cell necrosis were purchased from Selleck (Shanghai, China). And their working concertation according to the manufacturer’s instructions.

### RNA extraction and quantitative real-time PCR (qRT-PCR)

Total RNA extraction and reverse transcription were performed using trizol (Thermo Fisher Scientific) and Prime Script RT kits (Takara, Japan) according to the instructions. qRT-PCR was performed using SYBR GREEN PCR Master Mix (Takara, Japan). The relative levels of miRNA and circRNA were calculated using the 2^(-△△Ct)^ formulae. All the primers used in the study are shown in Table 1.

**TABLE 1:** Primer sequences.

Primer set	Forward primer	Reverse primer
let-7a-5p	CCC​CCC​TGA​GGT​AGT​AGG​TTG​TAT	CCA​GTG​CAG​GGT​CCG​AGG​T
let-7b-5p	CCC​CCT​GAG​GTA​GTA​GGT​TGT​GT	CCA​GTG​CAG​GGT​CCG​AGG​T
let-7c-5p	CCC​CCC​TGA​GGT​AGT​AGG​TTG​TAT	CCA​GTG​CAG​GGT​CCG​AGG​T
miR-98-5p	CCC​CCC​TGA​GGT​AGT​AAG​TTG​TAT	CCA​GTG​CAG​GGT​CCG​AGG​T
miR-202-3p	CCC​CAG​AGG​TAT​AGG​GCA​TG	CCA​GTG​CAG​GGT​CCG​AGG​T
miR-615-5p	GGGGGTCCCCGGTGCTCG	CCA​GTG​CAG​GGT​CCG​AGG​T
miR-19a-3p	CCC​TGT​GCA​AAT​CTA​TGC​AAA​A	CCA​GTG​CAG​GGT​CCG​AGG​T
U6	CGC​TTC​GGC​AGC​ACA​TAT​ACT​A	GGA​ACG​CTT​CAC​GAA​TTT​GC
SLC7A11	TTG​TTT​TGC​ACC​CTT​TGA​CAA​T	GAC​GAT​GCA​TAT​CTG​GGC​ATT
GAPDH	CAT​GTT​CGT​CAT​GGG​TGT​GAA	GGC​ATG​GAC​TGT​GGT​CAT​GAG
miR-615-5p-RT	GTC​GTA​TCC​AGT​GCA​GGG​TCC​GAG​GTA​TTC​GAC​TGG​ATA​CGA​CGA​TCC​G	
let-7a-5p-RT	GTC​GTA​TCC​AGT​GCA​GGG​TCC​GAG​GTA​TTC​GCA​CTG​GAT​ACG​ACA​ACT​AT	
let-7b-5p-RT	GTC​GTA​TCC​AGT​GCA​GGG​TCC​GAG​GTA​TTC​GCA​CTG​GAT​ACG​ACA​ACC​AC	
let-7c-5p-RT	GTC​GTA​TCC​AGT​GCA​GGG​TCC​GAG​GTA​TTC​GCA​CTG​GAT​ACG​ACA​ACC​AT	
miR-98-5p-RT	GTC​GTA​TCC​AGT​GCA​GGG​TCC​GAG​GTA​TTC​GCA​CTG​GAT​ACG​ACA​ACA​AT	
miR-202-3p-RT	GTC​GTA​TCC​AGT​GCA​GGG​TCC​GAG​GTA​TTC​GCA​CTG​GAT​ACG​ACT​TCC​CA	
miR-19a-3p-RT	GTC​GTA​TCC​AGT​GCA​GGG​TCC​GAG​GTA​TTC​GCA​CTG​GAT​ACG​ACT​CAG​TT	

### Western blotting analysis

Total proteins were extracted from SCC-25 or CAL-27 cells using RIPA lysis buffer (Beyotime Biotechnology, China). The protein concentration was measured using the BCA protein assay kit (Solarbio, China) according to the manufacturer’s instructions. Equal amounts of proteins were separated in 8%–12% sodium dodecyl sulfate-polyacrylamide gel and transferred to polyvinylidene fluoride membranes (PVDF). The membranes were blocked with 5% skim milk powder in PBST at room temperature for 2 h before overnight incubation at 4°C with the primary antibody. After three washes with PBS, the PVDF membrane was incubated with the secondary antibody for 2 h at room temperature. Finally, protein bands were visualized using the ChemiDoc Touch Imaging System (Biorad). The following antibodies were used in the Western blotting process: anti-GPX4 (1:1000, Abcam, cat.no.ab125066), SLC7A11 (1:1000, Abcam, cat.no.ab125066), GAPDH (1:2000, Elabscience, cat.no.E-AB-20059), and anti-Rabbit IgG (1:10,000, Proteintech, cat. no.10285-1-AP).

### Immunohistochemical (IHC) analysis

Oral tissues were sliced into thin (3–6 μm) sections, sequentially dewaxed with xylene and anhydrous ethanol, and then treated with citric acid antigen repair solution at high temperature. The tissue sections were then incubated overnight at 4°C with primary antibody diluted with PBS (1:100) and thereafter with DAB on and hematoxylin dye at room temperature. Finally, the tissue sections were dehydrated, and images were acquired using Olympus BX53 microscope (Olympus, Tokyo, Japan).

### Cell viability assay

Cell viability was used to measure the proliferation rates of cells. Here, 100 ul of treated cells were inoculated and cultured in 96-well plates for 24 h, and the proliferation rate of cells was detected using the Cell Counting Kit (CCK-8, Solarbio, China). The OD value was recorded at 450 nm by a microplate spectrophotometer (Molecular Devices, Sunnyvale, CA, United States).

### Cell death analysis

Treated cells (1×10^6^) were washed with PBS and resuspended in 100 μL 1 × Annexin V Binding Buffer. Annexin V (2.5 μL) and PI (2.5 μL) (Solarbio, China) were added to the cell suspension and incubated at room temperature for 15 min in darkness. Finally, 1 × Annexin V was added to the cell suspension to achieve a final volume of 500 μL before analysis with flow cytometry (Bechman Coulter, Palo, Alto, CA, United States).

### Luciferase reporter assay

Cells were transfected with wild-type (WT) or mutated (MUT) circ-CDK8 3′UTR reporter plasmid with miR-615-5p mimics or negative controls using Lipofectamine 3,000 according to the manufacturer’s instructions. After 48 h transfection, firefly and Renilla luciferase activities were measured consecutively using the Dual-Luciferase Reporter Assay System (Promega, United States).

### Wound healing and transwell assays

Cells were inoculated in six-well plates and treated differently. When the cell density reached 95% confluence, a scratch was made using a 200 ul gun tip. The cells were washed three times with PBS, and the culture medium without FBS was replaced. The cells were placed in the incubator and observed after 12 h and 24 h. The cells were digested, re-suspended in an FBS-free medium, and counted, and 1×10^5^ cells were added to the upper layer of the transwell chamber, which was placed in a 24-well plate. FBS-containing medium (600ul) was added to the lower layer. Cells that had migrated to the lower chamber after 24 h and 48 h were gently scrapped, fixed in 4% paraformaldehyde for 30 min, stained with 0.1% crystal violet for 30 min, washed with running water, and observed under a microscope.

### Transmission electron microscopy (TEM)

The SCC-25 and CAL-27 cells were harvested and fixed in the 2.5% Glutaraldehyde (Solarbio, China). The cells were then observed and photographed with a transmission electron microscope (JEM-1200, Jeol, Japan).

## Detection of intracellular ROS, MDA, and GSH

SCC-25 and CAL-27 cells were specially treated and inoculated onto cell culture plates. The level of ROS was determined using 2′7′-dichlorodihydrofluorescein diacetate (DCFH-DA), while malondialdehyde (MDA) and GSH levels were determined using respective kits (Beyotime Biotechnology).

### *In vivo* nude mice model

The protocols for animal experiments were approved by the Animal Care and Use Committee of the Affiliated Hospital of Qingdao University (AHQU-MAL20210604). Four-week-old female nude mice were purchased from the Charles River (Beijing, China). The animal experiments were conducted at the Affiliated Hospital of Qingdao University animals’ Laboratory. The mice were injected subcutaneously with sh-NC or sh-circ-CDK8 CAL-27 cells (5 × 10 ^6^) in 100 ul PBS. When the tumors reached about 80 mm^3^, the mice were injected intraperitoneally with 5 mg/kg erastin every 2 days eight times. The tumor size was analyzed every 5 days.

### Statistical analysis

Data were analyzed using GraphPad Prism 8. Differences between groups were analyzed using *t*-test. Continuous normally distributed data were expressed as mean ± SEM. *p < 0.05, **p < 0.01 were considered statistically significant.

## Results

### The expression of circ-CDK8 was higher in OSCC

**FIGURE 1:**
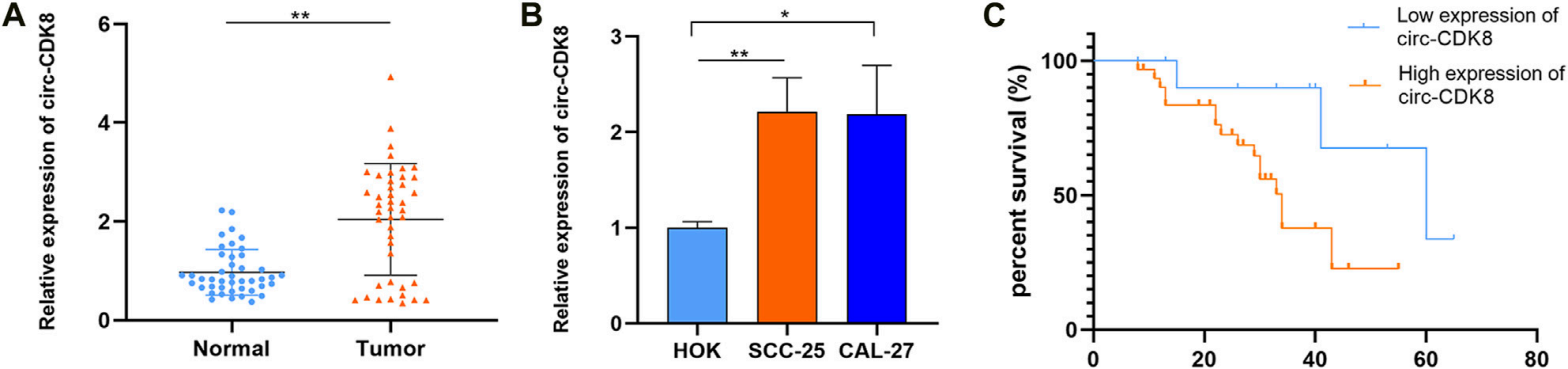
The expression of circ-CDK8 was increased in OSCC. **(A, B)** The expression of circ-CDK8 in tissues and OSCC lines was determined by qRT-PCR (n = 43). **(C)** The overall survival rate analysis in patients with OSCC using the Kaplan-Meier method.

Based on the findings of a previous study on circRNA (Gao, Zhao, Li, Dou, Wang, Liu, Ren and Zhi, 2019), we first evaluated the expression of circ-CDK8 in 43 OSCC tumor tissues and matched normal samples. We found that circ-CDK8 levels were significantly higher in OSCC tissues compared with the normal tissues (Figure 1A). We further investigated the clinicopathologic characteristics of OSCC patients and found that the expression of circ-CDK8 positively correlated with tumor size, lymph node metastasis and histological grade (Table 2). Accordingly, Kaplan-Meier analysis revealed that patients with higher circ-CDK8 levels had a poorer prognosis than those with lower expression (Figure 1C). Similarly, the expression of circ-CDK8 was higher in OSCC cells (SCC-25 and CAL-27) than in the human oral keratinocyte (HOK) (Figure 1B). These results showed that the expression of circ-CDK8 was different between OSCC and normal tissues. Thus, the distinct biological and clinical behaviours of OSCC cells may be implicated to circ-CDK8.

**TABLE 2:** The association of circ-CDK8 expression in forty-three OSCC patients with clinicopathologic characteristics.

	Circ-CDK8 expression			
Characteristics	Case number	Low	High	*p*-value
		(n = 12)	(n = 31)	
Gender				0.836
Male	24	7	17	
Female	19	5	14	
Age				0.173
≤60	26	5	20	
>60	17	7	11	
Tumor size				0.033*
≤3	21	9	12	
>3	22	3	19	
Lymph node metastasis				0.040*
N0	18	8	10	
N+	25	4	21	
Histological grade				0.003*
Well and moderately	20	10	10	
Poorly	23	2	21	

*p< 0.05.

### Circ-CDK8 regulated the biological behavior of OSCC cells

**FIGURE 2:**
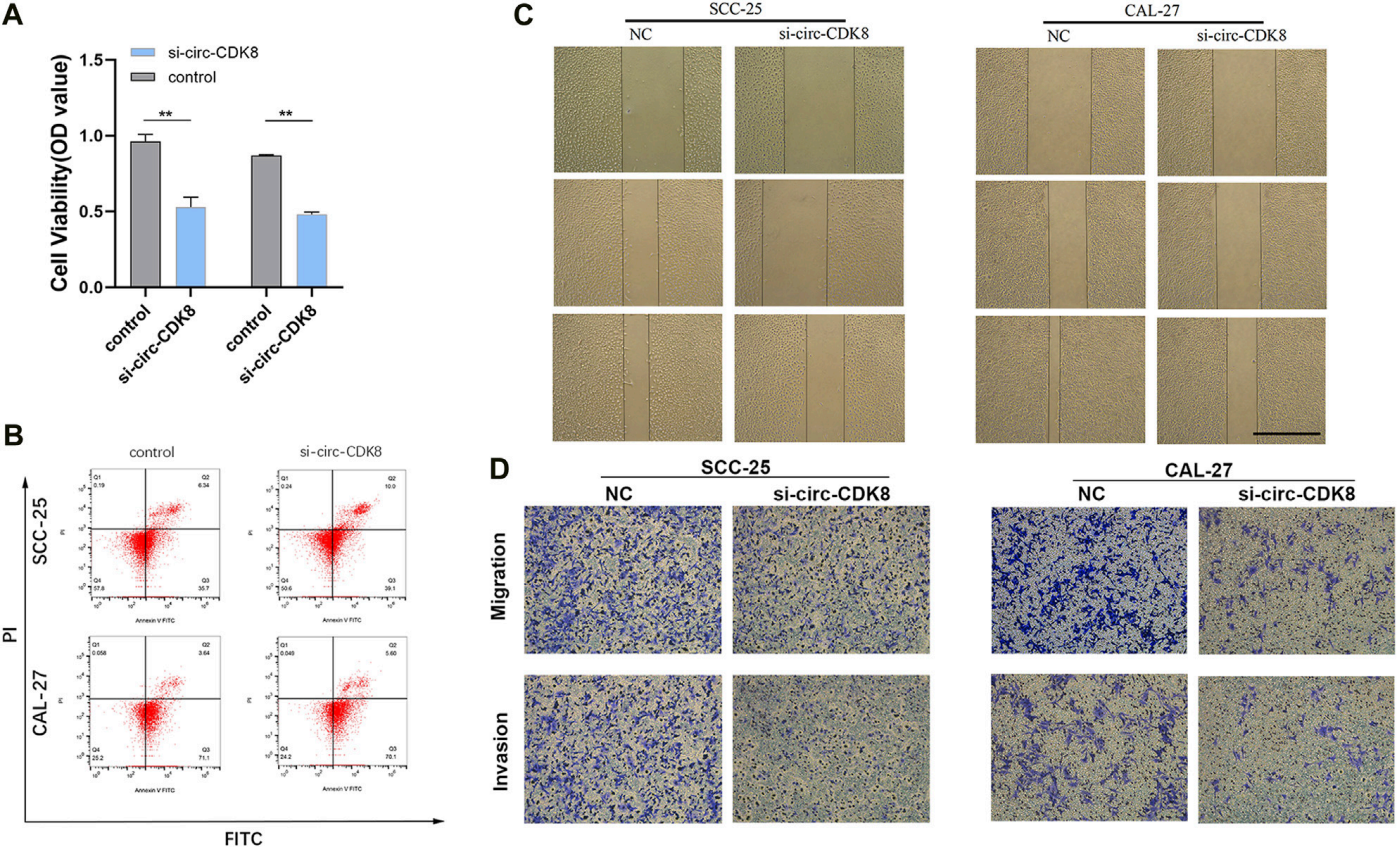
Interference with circ-CDK8 inhibits OSCC cell proliferation migration invasion. **(A)** CCK8 assay was used to detect cell viability of SCC-25 and CAL-27 transfection with si-circ-CDK8 and negative control (NC). **(B)** Flow cytometry showed that cell death rate was increased in si-circ-CDK8 cells. **(C, D)** Wound-healing assay and transwells assays revealed the migration and invasion of OSCC transfected with si-circ-CDK8 and NC. Scar bar = 1000 μm.

Since the biogenesis, function, and clinical significance of circRNA have been extensively studied (Li et al., 2020a), we further explored whether circ-CDK8 regulates the biological behavior of OSCC cells. We found that low circ-CDK8 levels significantly decreased the proliferation of SCC-25 and CAL-27 cells (Figure 2A). Moreover, the rate of cell death was significantly higher in the circ-CDK8 interference group than in the control group (Figure 2B). Reducing the circ-CDK8 level slowed down the migration of OSCC cells significantly (Figure 2C). Similarly, we found that the migration and invasion of cells was significantly lower in the circ-CDK8 interference group (Figure 2D). Generally, circ-CDK8 was highly expressed in oral squamous carcinoma tissues and interfering with circ-CDK8 transcription significantly inhibited cell proliferation, migration and invasion of OSCC cell lines.

### MiR-615-5p and SLC7A11 are downstream genes regulated by circ-CDK8

**FIGURE 3:**
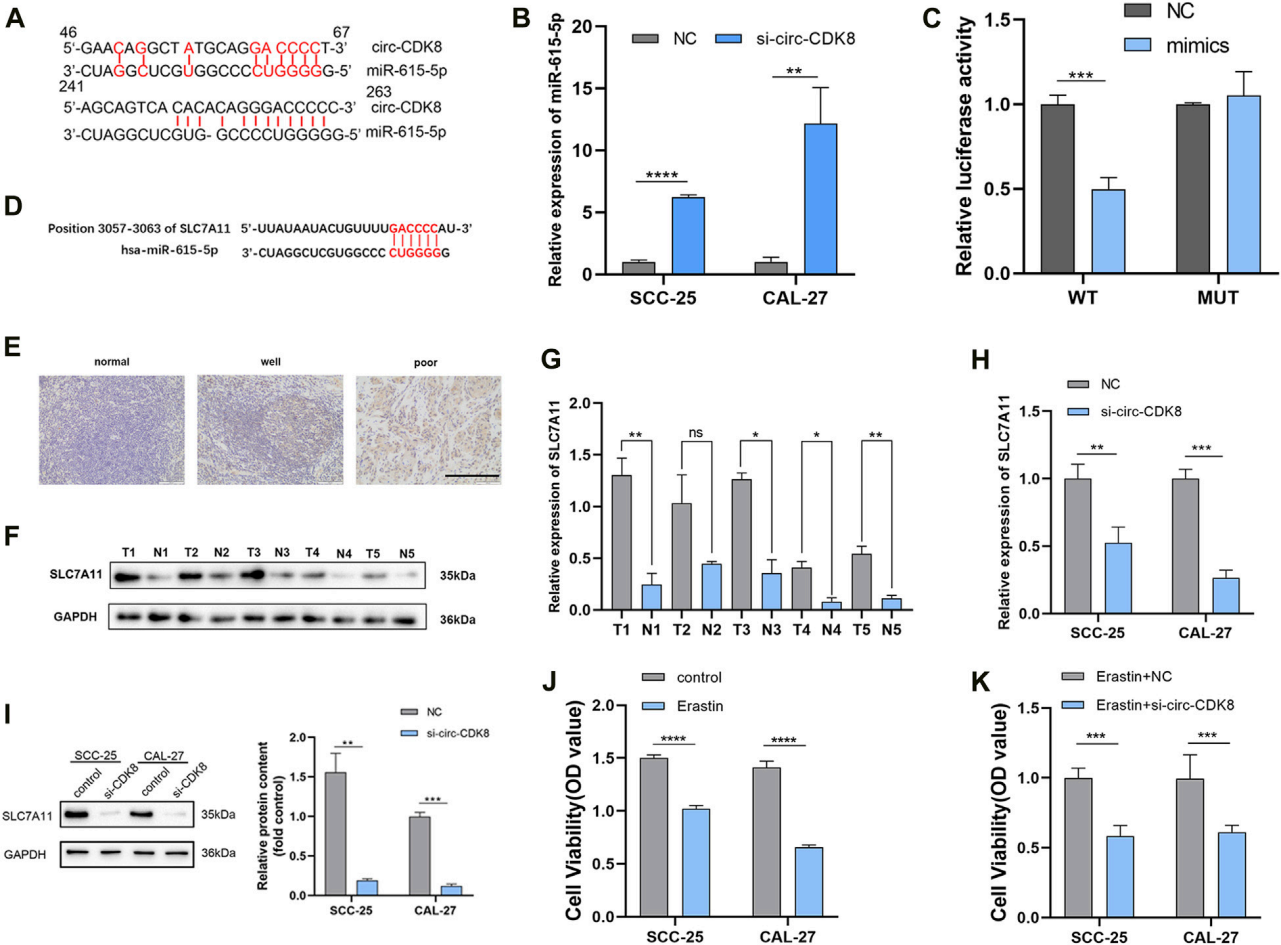
circ-CDK8 is a molecular sponge for miR-615-5p. **(A)** A schematic graph of the target miRNAs-binding sequence. **(B)** The mRNA expression results of miRNAs measured using qRT-PCR in SCC-25 and CAL-27 cells stably transfected with si-circ-CDK8. **(C)** The luciferase activity reporter assay results showed the miR-615-5p binding site on circ-CDK8. **(D)** Illustration of the base pairing between miR-615-5p and SLC7A11. **(E)** IHC staining for SLC7A11 are shown at ×200 magnification, scale bar = 100 µm. **(F, G)** Five paired samples of OSCC tissues (T) and adjacent normal tissues (N) were analyzed by Western blot. **(H)** The mRNA expression results of SLC7A11 measured using qRT-PCR in SCC-25 and CAL-27 cells stably transfected with si-circ-CDK8. **(I)** The protein levels of SLC7A11 in SCC-25 and CAL-27 cells transfected with si-circ-CDK8 and NC. **(J, K)** CCK8 assay was used to detect cell viability of SCC-25 and CAL-27 transfection with si-circ-CDK8 and NC under the treatment of Erastin. Scar bar = 400 μm.

The downstream targets of circ-CDK8 were identified using bioinformatics analyses using data in the Starbase v2.0 database. The results showed that let-7a-5p, let-7b-5p, let-7c-5p miR-98-5p, miR-202-3p, miR-615-5p, miR-19a-3p could be downstream targets of circ-CDK8. qRT-PCR analyses further revealed that overexpression of circ-CDK8 significantly reduced the expression of let-7b-5p and miR-615-5p (Supplementary Figure S1). Thus, miR-615-5p expression was analyzed in the subsequent experiments. Previous studies have shown that circRNAs function by sponging miRNAs(Hong et al., 2020). Firstly, we found circ-CDK8 and miR-615-5p gene targets (Figure 3A). Further analysis revealed that interference with circ-CDK8 in both SCC-25 and CAL-27 cell lines (Figure 3B). To further confirm the correlation between circ-CDK8 and miR-615-5p expression, miR-615-5p mimics was co-transfected in the SCC-25 cells with circ-CDK8 wild type (WT) or mutated (MUT) circ-PKD2 (Figure 3C). These results suggested that circ-CDK8 acted as a miR-615-5p sponge in OSCC. Then we searched for downstream miR-615-5p target genes through the TargetScan database and found that miR-615-5p targets SLC7A11 (Figure 3D). We analyzed the expression of SLC7A11 in various tumors through Timer database (https://cistrome.shinyapps.io/timer/), and results showed that SLC7A11 expression was significantly difference in head and neck squamous cell carcinoma (HNSC) (Supplementary Figure S3). In addition, we downloaded phenotypic data of head and neck squamous cell carcinoma (HNSCC) from the TCGA website. We preliminarily analyzed the expression level of SLC7A11 and clinicopathological indicators, and found that the expression of SLC7A11 was related to gender (Supplementary Table S4). Immunohistochemical results showed that compared with the normal tissues, SLC7A11 was highly expressed in tumour tissues (Figure 3E). Western blot and qRT-PCR revealed similar results (Figures 3F, G). QRT-PCR and Western blot were performed to confirm the regulatory relationship between circ-CDK8 and SLC7A11. The results showed that interference with circ-CDK8 significantly reduced SLC7A11 expression at both mRNA and protein levels (Figures 3H, I). Since SLC7A11 is a critical regulator of ferroptosis (Lang et al., 2019), we speculated that circ-CDK8 regulated ferroptosis. In the present study, ferroptosis was induced using erastin. Results showed that erastin treatment reduced the viability of both SCC-25 and CAL-27 had reduced cell viability under Erastin treatment, suggesting that OSCC cells are sensitive to ferroptosis (Figure 3J). Importantly, interfering with circ-CDK8 in Erastin-treated SCC-25 and CAL-27 cells still reduced cell viability (Figure 3K), suggesting a link between circ-CDK8 and ferroptosis. Generally, our results showed that circ-CDK8 regulates miR-615-5p transcription and translation, and it plays an important role in OSCC cells’ ferroptosis.

### Circ-CDK8 regulates ferroptosis of OSCC cells

**FIGURE 4:**
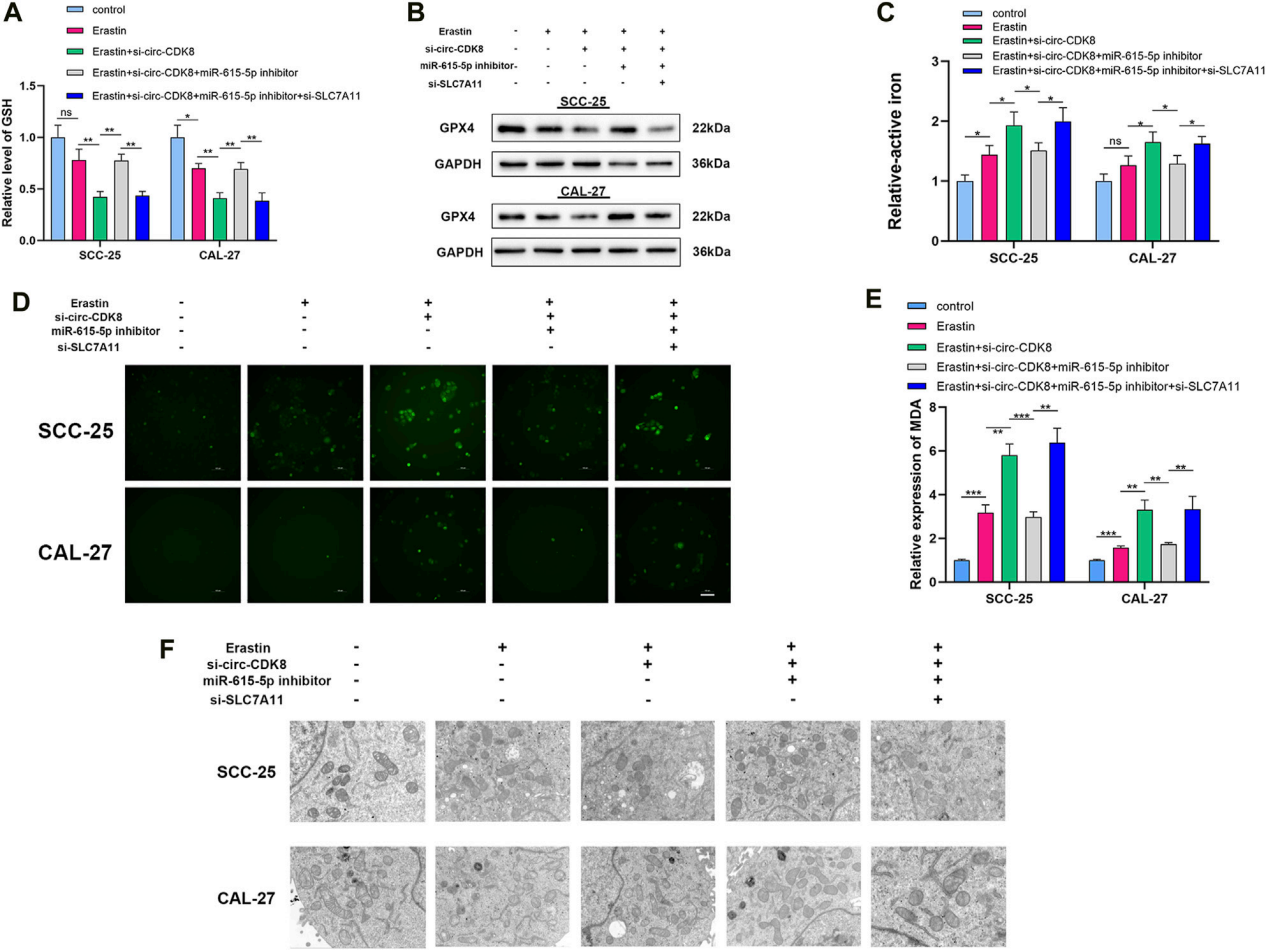
circ-CDK8 regulated ferroptosis of OSCC. **(A)** Intracellular GSH in different groups of SCC-25 and CAL-27 cells, cells transfected with si-circ-CDK8, miR-615-5p inhibitor and/or si-SLC7A11. **(B)** Western blot analysis of GPX4 expression in different groups. **(C)** Intracellular ferrous iron was showed in different groups. **(D)** Fluorescence microscopy to detect ROS levels in different groups. **(E)** The content of MDA in different groups in SCC-25 and CAL-27 cells. **(F)** TEM analysis was performed to evaluate alterations in mitochondrial morphology in OSCC cells. Scar bar = 1 μm.

Because circ-CDK8 has been linked to ferroptosis, we investigated the mechanism by which circ-CDK8 regulates ferroptosis. Previous studies have shown that GPX4 is a key negative regulatory enzyme for the onset of ferroptosis and that GPX4 deficiency leads to lipid peroxidation and ferroptosis. In addition, a significantly low GSH level was associated with GPX4 deficiency and ROS accumulation (Yang et al., 2014). Interestingly, our results showed that miR-615-5p knockdown increased the expression of GSH and GPX4 by reducing the inhibition of ferroptosis by circ-CDK8 under erastin treatment (Figures 4A, B). Similarly, circ-CDK8 significantly increased the levels of active iron, but this effect was attenuated by miR-615-5p (Figure 4C). In addition, reactive oxygen (ROS), lipid peroxides and malondialdehyde (MDA) were higher in the si-circ-CDK8 group than in the miR-615-3p inhibitor group (Figure 4D, Supplementary Figure S2; Figure 4E). Moreover, mitochondrial swelling and mitochondrial cristae reduction or disappearance were observed in the circ-CDK8 interference group. However, miR-615-5p inhibition reversed this phenomenon (Figure 4F). Overall, circ-CDK8 regulated ferroptosis by sponging miR-615-5p and inhibiting the ability of SLC7A11, and mitochondria played a critical role in the ferroptosis of OSCC cells.

### Circ-CDK8 impacted the biological behavior of OSCC cells by regulating SLC7A11 expression

**FIGURE 5:**
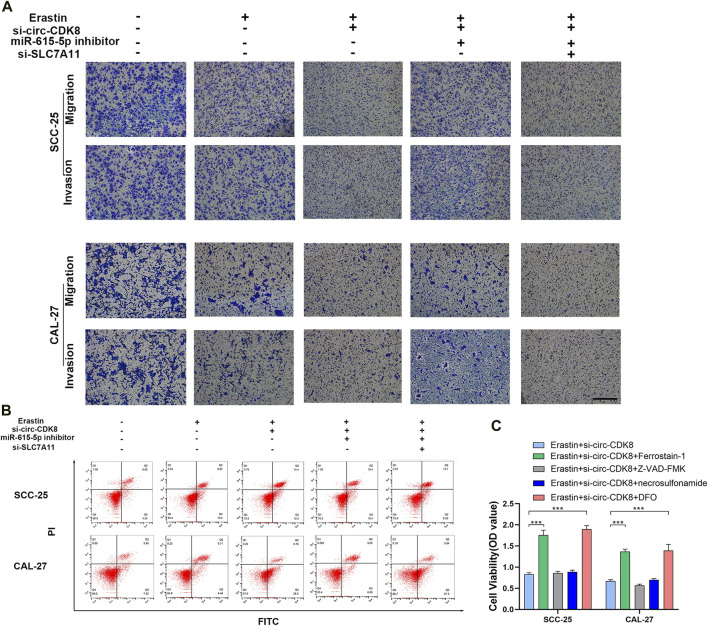
circ-CDK8 regulated OSCC cells biological behavior through miR-615-5p/SLC7A11. **(A)** Transwells assays revealed the migration and invasion of OSCC transfected with si-circ-CDK8, miR-615-5p inhibitor and/or si-SLC7A11 under the Erastin. Scar bar = 100 μm. **(B)** Flow cytometry showed that cell death rate in different. **(C)** Cell viability of SCC-25 cells following treatment with ferrostain-1 (0.5uM), deferoxamine (DFO, 100 µM) or Z-VAD-FMK (20uM) for 12 h.

As a member of the glutamate/cystine reverse transport system (system Xc-), SLC7A11 suppresses the development/progression of several cancers (He et al., 2023; Xu et al., 2023). In the study, modulating SLC7A11 expression reduced the migration and invasion ability of SCC-25 and CAL-27 cells. Interestingly, co-transfection with miR-615-5p inhibitor and si-SLC7A11 augmented the inhibition of the migration and invasion ability of SCC-25 and CAL-27 cells (Figure 5A). Similarly, interfering with the circ-CDK8 induced the cell death of SCC-25 and CAL-27, and co-transfection with miR-615-5p inhibitor increased the rate of cell death (Figure 5B). Interestingly, we found that only ferroptosis inhibitors (Ferropstain-1, DFO) reversed the reduction in cell viability induced by Erastin, whereas apoptosis and necrosis inhibitors did not (Figure 5C). Based on the above results, SLC7A11 plays an important role in the migration and invasion of OSCC cells by inhibiting ferroptosis, and circ-CDK8 acts via the miR-615-5p pathway.

### Circ-CDK8 regulated OSCC cell growth *in vivo*

**FIGURE 6:**
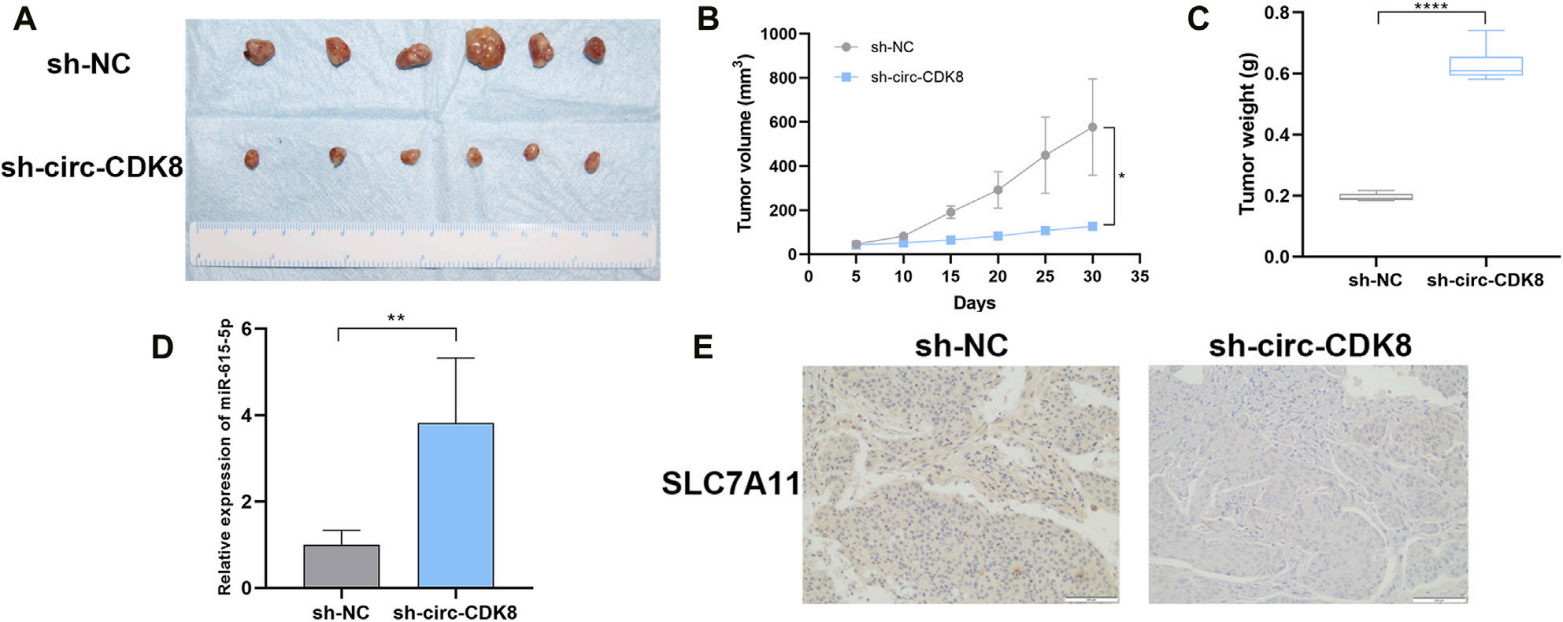
Effect of interference with circ-CDK8 on tumorigenesis *in vivo*. **(A–C)** The tumor size and tumor weight were observed in two groups. **(D)** The expression of miR-615-5p. **(E)** The expression of SLC7A11 was observed between the two groups. Scar bar = 400 μm.

To further explore whether circ-CDK8 regulates cancer cell growth *in vivo*, SCC-25 cells transfected with sh-circ-CDK8 or negative control were subcutaneously injected into nude mice. We found that the volume and weight of the SCC-25 xenograft decreased in the sh-circ-CDK8 group (Figures 6A–C). The expression of miR-615-5p was upregulated in sh-circ-CDK8 groups (Figure 6D). Immunohistochemistry (IHC) staining showed that the expression of SLC7A11 was lower in the sh-circ-CDK8 group (Figure 6E). These *in vivo* results further confirmed that interfering with circ-CDK8 inhibits the OSCC proliferation.

## Discussion

**FIGURE 7:**
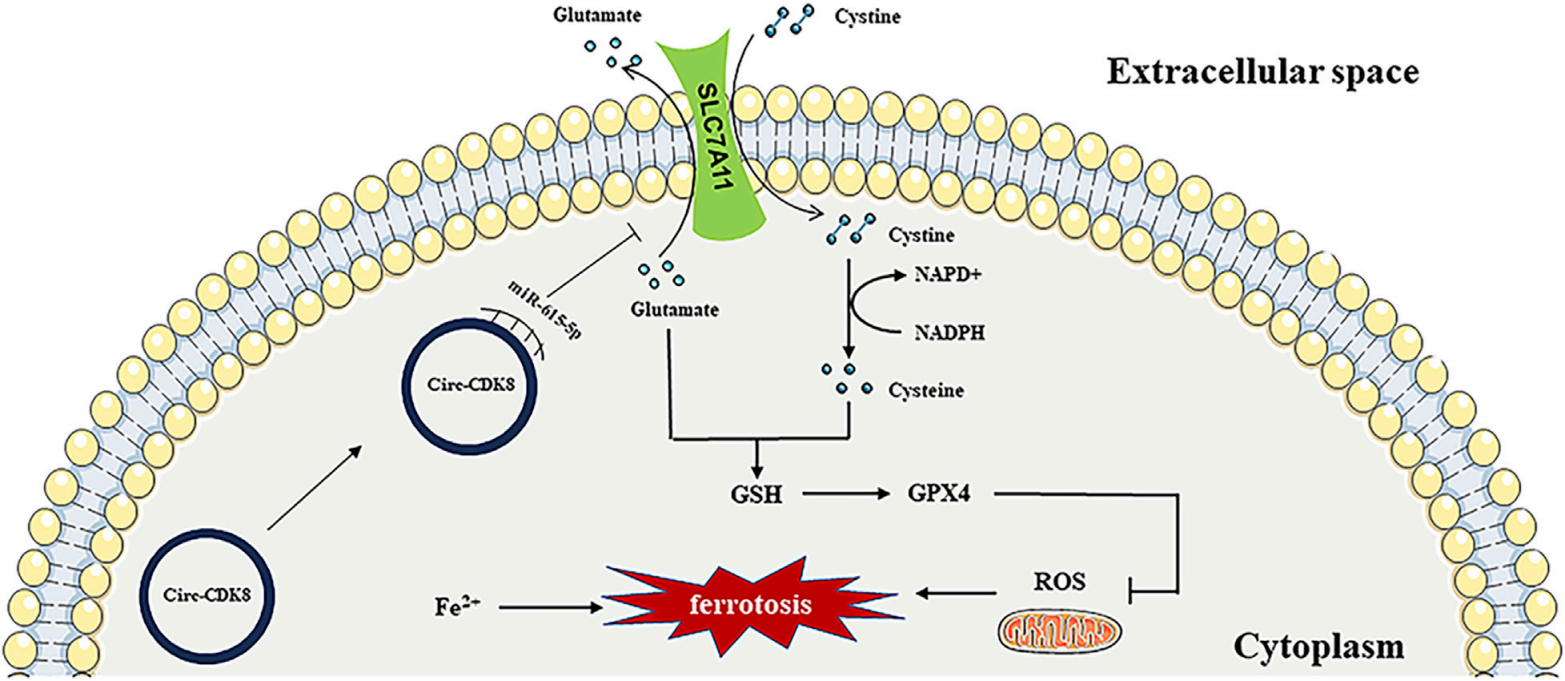
The mechanisms of circ-CDK8 regulating ferroptosis.

OSCC is the most prevalent oral malignancy. Currently, OSCC is mainly treated by surgery, supplemented with radiotherapy. With the continuous progress in diagnostic imaging technology, surgical methods, radiotherapy and other adjuvant treatments, as well as systemic treatments, the mortality rate of oral cancer has been decreasing. However, the 5-year survival rate of patients with squamous cell carcinoma of the oral cavity is only about 40%–60% (Mody, Rocco, Yom, Haddad and Saba, 2021). Recent studies have focused on the pathogenesis and markers of OSCC. The present study demonstrated that circ-CDK8 promotes the SLC7A11 expression by competitive sponging of miR-615-5p to prevent the ferroptosis of OSCC cells (Figure 7).

Accumulating evidence has shown that abnormal expression circRNAs play a critical role in cancer development (Bose and Ain, 2018; Li et al., 2023; Zhang et al., 2023). In the study, circRNA microarrays revealed that circ-CDK8 is over-expressed in OSCC cells. Prior to this study, the role of circ-CDK8 in cancer cells had not been reported. We found that circ-CDK8 expression was significantly higher in cancer tissues and OSCC cells than in normal tissue and HOK cells. The expression level of circ-CDK8 is tightly associated with clinical characteristics, such as TNM stage, histological grade, tumor size and postoperative survival rate of OSCC patients. Interfering with circ-CDK8 transcription/function significantly reduced the viability and migration of OSCC cells *in vivo* and *in vitro* (Figures 2A, 6A). Our findings revealed that circ-CDK8 is a critical tumor-promoting factor, and it regulates protein expression by modulating RNA-protein interactions or through RNA splicing.

To clarify the specific mechanism by which circ-CDK8 regulates OSCC progression, we predicted the possible gene targets of circ-CDK8 through bioinformatics analysis. We found that circ-CDK8 possibly targets let-7a-5p, let-7b-5p, let-7c-5p miR-98-5p, miR-202-3p, miR-615-5p, and miR-19a-3p. Studies (Wu M. et al., 2021; Zeng et al., 2021) have shown that the expression of miR-615-5p in OSCC cells is associated with the circ- CDK8. In our study, we found that interfering with miR-615-5p transcription/function can effectively abrogate the inhibition of cell migration invasion due to interference with circ-CDK8 function (Figure 5A). Our results demonstrated that miR-615-5p was a direct downstream target of circ-CDK8, and miR-615-5p effectively inhibited the migration and invasion of OSCC cells.

Further analysis of data in the TargetScan database revealed that SLC7A11 targets miR-615-5p. The main role of SLC7A11 is to mediate the transmembrane exchange of cystine and glutamate, which involves both the regulation of extracellular glutamate concentration and the provision of raw material for intracellular GSH synthesis (Lin et al., 2020; Yuan et al., 2021). GSH and GPX4 are active substances that inhibit the production of ROS in cells. In this study, we found that circ-CDK8 increased the expression of GSH and GPX4 by competitive sponging miR-615-5p. Meanwhile, inhibited the migration of SLC7A11 cells. Our study also confirmed that circ-CDK8 effectively decreases ROS production and promotes lipid peroxide accumulation, inhibiting the ferroptosis of OSCC cells. Whether circ-CDK8 affects the migration and invasion of OSCC cells by regulating ferroptosis was explored further.

Ferroptosis is a new type of regulated cell death, which differs from apoptosis and necrosis and is characterized by the accumulation of iron-dependent lipid peroxidation (Dixon et al., 2012). As the molecular and physiological mechanisms of ferroptosis continue to be elucidated, the strategy of circRNAs to treat tumors through ferroptosis has gained widespread support. For example, circRAPGEF5 medicated ferroptosis by modulating alternative splicing of TFRC in endometrial cancer, and circRAPGEF5/RBFOX2 axis might be a cancer therapy target (Zhang et al., 2022). CircEPSTI1 promotes cells progression by miR-375/409-3P/515-5p-SLC7A11 axis in cervical cancer (Wu P. et al., 2021). In the present study, we found that interfering with circ-CDK8 function decreased the expression of SLC7A11 and promoted the accumulation of MDA (end product of lipid peroxidation) and ROS under the treatment of si-circ-CDK8, inhibiting the migration and invasion of OSCC. Erastin, a laboratory ferroptosis inducer, produced significant inhibition of OSCC, that suggested that OSCC are sensitive to ferroptosis. Ferroptosis-related clinical chemotherapeutic agents such as sorafenib and sulfasalazine may play an important role in OSCC treatment. Our findings further revealed that circ-CDK8 increased the expression of SLC7A11, GSH and GPX4, which decreased the intracellular MDA and ROS levels. Additionally, circ-CDK8 decreased ferroptosis but increased the migration and invasion of OSCC cells. The current study shows, for the first time, that si-circ-CDK8 enhanced the sensitivity of OSCC cells to erastin-induced ferroptosis by regulating SLC7A11 *in vivo* and *in vitro*. Therefore, induction of ferroptosis by controlling the circ-CDK8/miR-615-5p/SLC7A11 regulatory axis might be a new therapeutic option for OSCC. In addition, with the wide application of cisplatin and iron death drugs in OSCC. It is more necessary to construct ferroptosis resistance cell lines and explore the regulation of circ-CDK8 on ferroptosis and biological behavior for it. Furthermore, interfering with the circ-CDK8 caused mitochondrial swelling and mitochondrial cristae reduction or disappearance in the OSCC cell line (Figure 4F). Therefore, targeting mitochondria-related ferroptosis could be a potential strategy for cancer therapy in the future.

In conclusion, our study first suggested that circ-CDK8 is over-expressed in oral squamous carcinoma cells and tissues. Furthermore, circ-CDK8 suppressed ferroptosis of OSCC cells through the miR-615-5p/SLC7A11 axis. These findings provided a novel insight into the molecular mechanisms of OSCC progression and presented a potential OSCC treatment strategy.

## Data Availability

The original contributions presented in the study are included in the article/Supplementary Material, further inquiries can be directed to the corresponding authors.
