# Supplementary material for: Circ-CDK8 regulates SLC7A11-mediated ferroptosis by inhibiting miR-615-5p to promote progression in oral squamous cell carcinomas
Source: Front Pharmacol. 2024 Aug 7;15:1432520. doi: 10.3389/fphar.2024.1432520 (PMC11335485; doi:10.3389/fphar.2024.1432520)
Supplement: Supplementary file 1 [file Table1.DOCX]

**Supplement 4. The association of circ-CDK8 expression in TCGA database with clinicopathologic characteristics**

|  |  | HIGH | LOW | X^2^ | *p* |
| --- | --- | --- | --- | --- | --- |
| Age | <60 | 43 | 39 |  |  |
|  | ≥60 | 40 | 43 | 0.298 | 0.585 |
| Alcohol | Yes | 59 | 59 |  |  |
|  | No | 24 | 23 | 0.015 | 0.902 |
| Site | Oropharynx  And laryngopharynx | 29 | 25 |  |  |
|  | Oral | 54 | 57 | 0.371 | 0.542 |
| Gender | Male | 73 | 57 |  |  |
|  | Female | 10 | 25 | 8.392 | 0.004* |
| Lymphovascular invasion present | Yes | 27 | 30 |  |  |
|  | No | 56 | 52 | 0.300 | 0.584 |
| Margin status | Yes | 14 | 9 |  |  |
|  | No | 69 | 73 | 1.194 | 0.275 |
| N | Yes | 44 | 46 |  |  |
|  | No | 39 | 36 | 0.158 | 0.691 |
| T | T1-T2 | 18 | 24 |  |  |
|  | T3-T4 | 65 | 58 | 1.250 | 0.264 |
| Stage | I-II | 11 | 15 |  |  |
|  | III-IV | 72 | 67 | 0.789 | 0.374 |
| Radiation  therapy | Yes | 55 | 62 |  |  |
|  | No | 28 | 20 | 1.746 | 0.186 |

**p*<0.05
