# Supplementary figures and images for: Circ-CDK8 regulates SLC7A11-mediated ferroptosis by inhibiting miR-615-5p to promote progression in oral squamous cell carcinomas
Source: Front Pharmacol. 2024 Aug 7;15:1432520. doi: 10.3389/fphar.2024.1432520 (PMC11335485; doi:10.3389/fphar.2024.1432520)

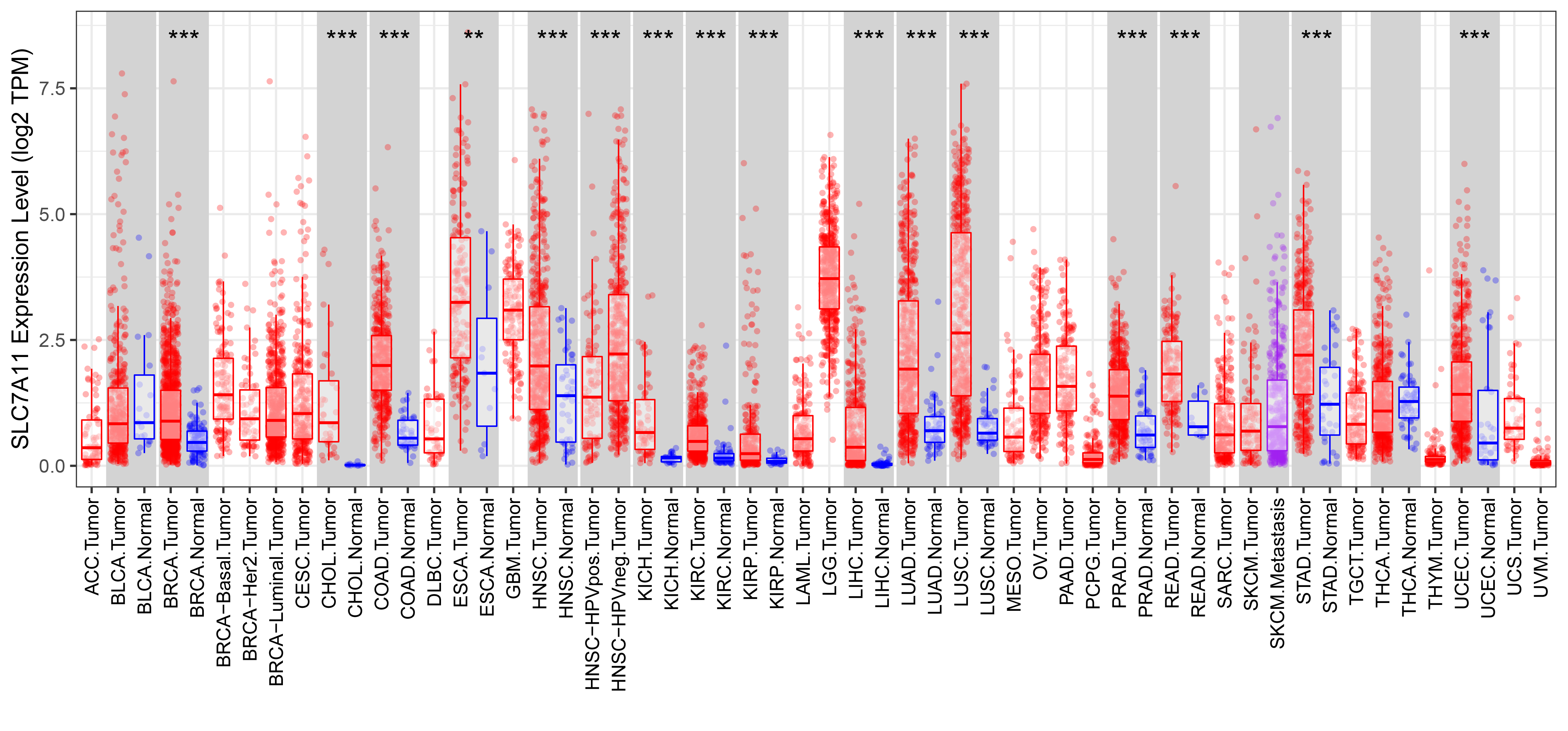

Supplement: Supplementary file 2 [file Image3.TIF]

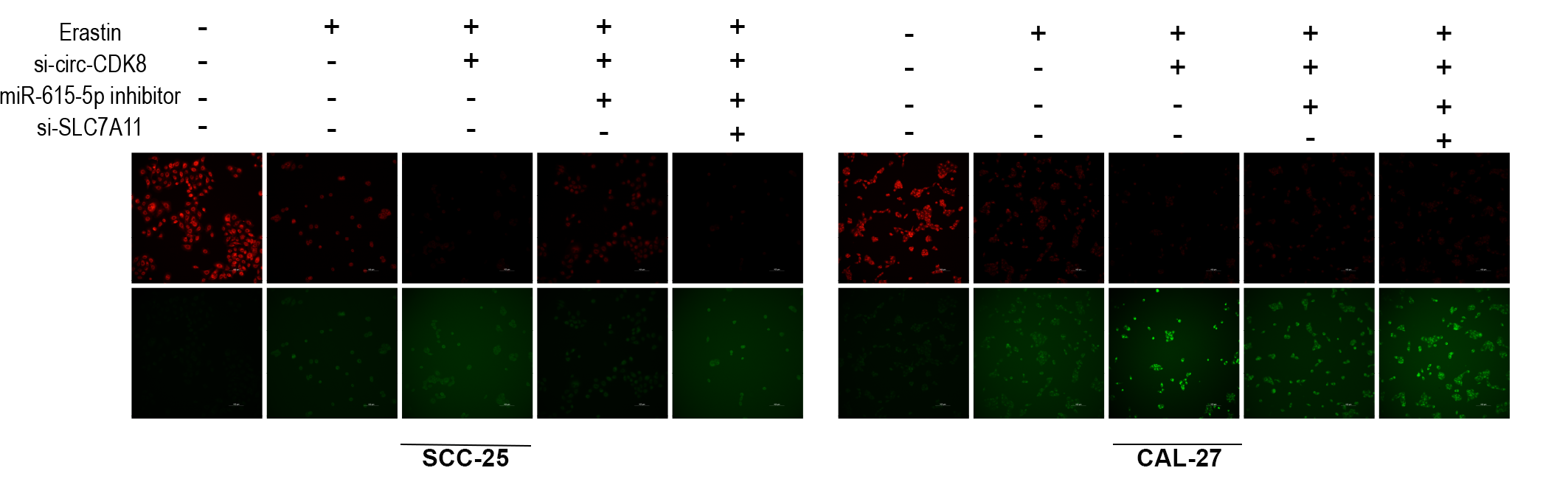

Supplement: Supplementary file 3 [file Image2.TIF]

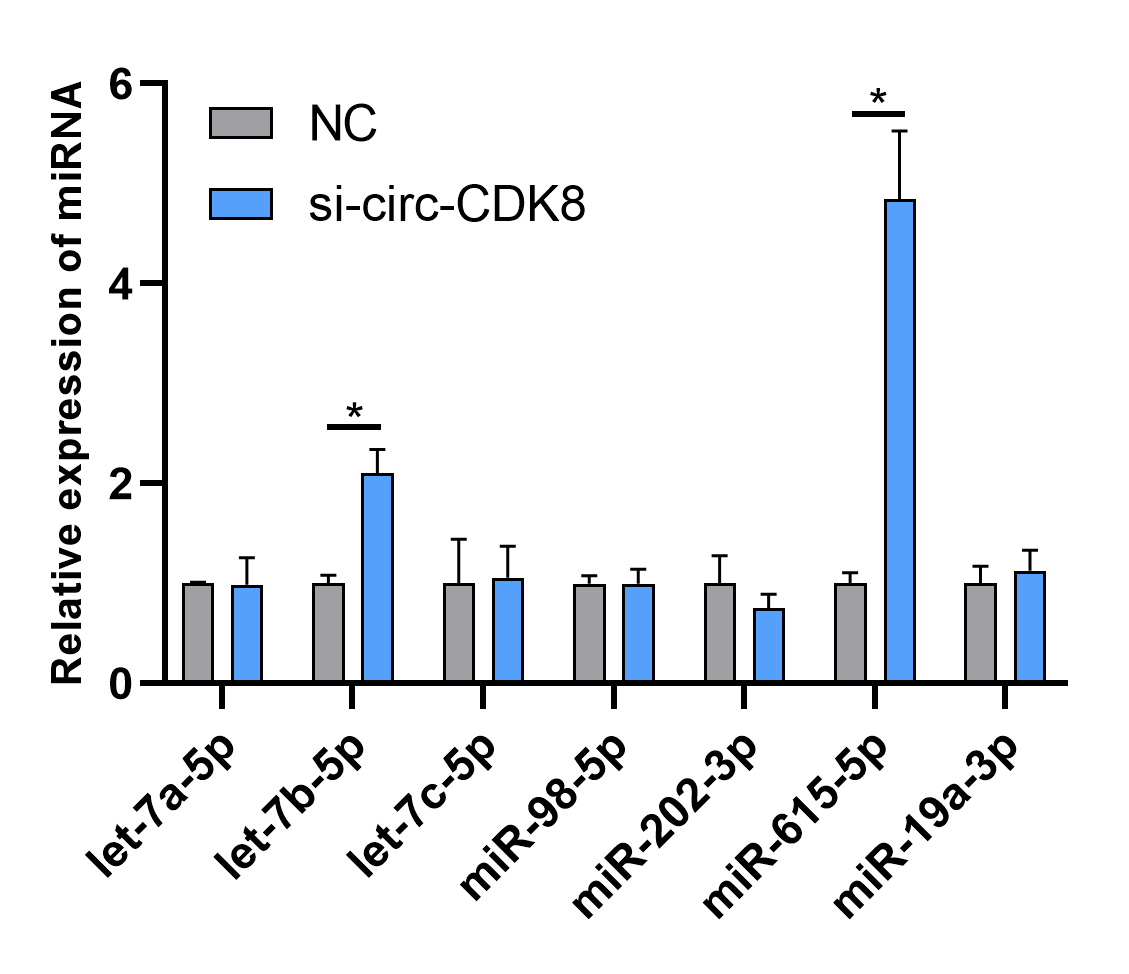

Supplement: Supplementary file 4 [file Image1.TIF]
